# Associations of Maternal Breastmilk microRNAs and Infant Obesity Status at 1 Year

**DOI:** 10.3390/genes15060813

**Published:** 2024-06-20

**Authors:** Emily Van Syoc, Molly Stegman, Rhea Sullivan, Alexandra Confair, Kaitlyn Warren, Steven D. Hicks

**Affiliations:** 1 Department of Biology, The Pennsylvania State University, University Park, PA 16802, USA; vansyoc@psu.edu; 2 One Health Microbiome Center, The Pennsylvania State University, University Park, PA 16801, USA; 3 Department of Pediatrics, Penn State College of Medicine, Hershey, PA 17033, USArsullivan2@pennstatehealth.psu.edu (R.S.);; 4 Department of Emergency Medicine, Boston Children’s Hospital, Boston, MA 02115, USA

**Keywords:** maternal breastmilk microRNA, milk consumption, infant obesity, microribonucleic acid, biomarker

## Abstract

Infant consumption of human milk (HM) is associated with a reduced risk of overweight and obesity, but the reasons for this relationship are not completely understood. There is emerging evidence that micro RNAs (miRNAs) regulate infant development and metabolism, but the associations between HM miRNAs and infant growth remain poorly understood. We examined the relationship between HM miRNA consumption and infant obesity in 163 mother–infant dyads to determine (1) if miRNA profiles differentiate infants with obesity, and (2) if individual miRNAs accurately predicted infant obesity status at one year of age. Infant obesity was categorized as weight-for-length (WFL) Z scores or conditional weight gain (CWG) in the 95th percentile. HM miRNA profile was associated with infant age (r^2^ = 6.4%, *p* = 0.001), but not maternal obesity status (r^2^ = 1.5%, *p* = 0.87) or infant weight status (WFL Z-score) at birth (r^2^ = 0.6%, *p* = 0.4), 1 month (r^2^ = 0.5%, *p* = 0.6), or 4 months (r^2^ = 0.8%, *p* = 0.2). Nine HM miRNAs were associated with either 12-month CWG or 12-month WFL Z scores. Among these 9 miRNAs, miR-224-5p remained significant in a logistic regression model that accounted for additional demographic factors (estimate = −27.57, *p* = 0.004). These findings suggest involvement of HM miRNAs and particularly miR-224-5p in infant growth, warranting further investigation. To our knowledge, this is the largest study of HM miRNAs and early-life obesity and contributes to the understanding of the relationship between HM miRNAs and infant growth.

## 1. Introduction

Childhood overweight and obesity have emerged as a major global health crisis, with 340 million children and adolescents aged 5 to 19 being overweight or obese in 2016 [1]. The prevalence of childhood obesity has been on the rise for the past few decades, climbing from 4% in 1975 to 18% in 2016 [1]. Childhood overweight and obesity often persist into adulthood and have been linked to co-morbidities including hypertension, coronary heart disease, type 2 diabetes, and insulin sensitivity [2]. Early intervention is essential for the prevention of obesity, but relies on identifying children who are at an increased risk of obesity as adults. Existing statistical models use risk factors such as maternal body mass index (BMI) and infant birth weight to predict being overweight or obesity in childhood, but limitations in validation and non-standard reporting prevents the widespread implementation of these models [3].

The infant growth rate during the first few months or years of life is associated with childhood obesity and may have predictive value [4,5,6,7]. In one study, the rate of weight gain in infancy better predicted being overweight later in childhood than the weight of the parents [8]. Rapid weight gain during infancy has emerged as a strong risk factor, and possible predictive method, for obesity in childhood and adulthood [9,10,11]. Given that infant weight gain may predict childhood overweight and obesity with implications for lifelong disease risk, it is important to better understand the factors affecting weight gain in infancy including regulatory molecules found in human milk.

Human milk (HM) is widely recognized as the optimal source of infant nutrition [12,13]. HM is key a source of macronutrients and bioactive factors, such as insulin, leptin, ghrelin, and microRNAs (miRNAs), which can regulate the growth and development of breastfeeding infants [14,15,16]. miRNAs are small non-coding RNA molecules in HM and serve a variety of regulatory functions in immunity, metabolism, and development [17,18,19,20,21]. However, the association between HM microRNAs and infant growth is an area of active investigation [22,23,24,25,26]. Recent studies have examined the relationship between several HM miRNAs and infant body composition within the first 6 months of life, and found that certain miRNAs were associated with infant weight [26], fat mass, and fat-free mass [27], suggesting that miRNAs may play a role in infant growth and body composition. However, the limitations of these studies include small sample sizes, analyses restricted to few miRNAs, and inconsistent HM sampling over time, necessitating research with larger sample sizes, untargeted quantification of the entire miRNA profile, and time-series sampling to investigate how the HM miRNA landscape evolves with infant growth.

The goal of this study was to explore associations between HM miRNAs and early-life obesity. We conducted an analysis of mother–infant dyads with HM collected at three timepoints (infant birth, 1 month, and 4 months) and corresponding anthropometric data through the first year of life. We sought to determine if HM miRNAs early in development could predict obesity later in infancy, and if the predictive utility of miRNAs was equal to or greater than previously known demographic associations (i.e., weight-for-length, conditional weight gain) of infant obesity. To our knowledge, this is the largest study of HM miRNAs and early-life obesity.

## 2. Materials and Methods

### 2.1. Participants

There were 221 mother–infant dyads enrolled in this study. Inclusion criteria were mothers who delivered at term (>35 weeks) and intended to feed their infant HM beyond 4 months. Exclusion criteria were (1) maternal morbidities that could impact lactation or milk miRNAs (i.e., cancer, drug addiction, HIV infection); (2) plan for infant adoption; (3) presence of neonatal conditions that would affect the ability to breastfeed; (4) plan for pediatric care outside of Penn State Health; and (5) non-English speaking. Details on recruitment and enrollment locations have been previously described [22]. This study was approved by the Penn State College of Medicine Institutional Review Board (protocol STUDY00008657) and registered at ClinicalTrials.gov as NCT04017520. All participants gave written informed consent.

### 2.2. HM Sample Collection

As previously described [22], HM was manually expressed into RNAse-free tubes from a sterilized nipple surface. Foremilk (pre-feed) samples were collected at infant birth, 1 month, and 4 months of age. These timepoints were selected to reflect the evolution of milk miRNA concentrations that occur throughout lactation [17]. To control for differences between breasts, mothers utilized the same breast for each collection. Time of collection was recorded for all samples to ensure that daily variations in milk miRNA concentrations did not contribute to between-group differences. There were 163 mothers who completed the study and 58 mothers who were excluded due to failure to provide an HM sample (*n* = 29) or complete longitudinal surveys about infant feeding patterns (*n* = 29). The 163 mothers contributed 442 human milk samples across the 3 timepoints. Specifically, there were 127 mothers that contributed 3 samples, 25 mothers that contributed 2 samples, and 11 mothers that gave 1 sample. Collected HM samples were immediately stored at −20 °C and moved to long-term storage at −80 °C within 4 weeks. Samples underwent only 1 freeze–thaw cycle prior to RNA extraction.

### 2.3. Sample and Data Processing

HM RNA was processed as previously described [22]. Briefly, HM samples were separated into lipid and aqueous fractions via centrifugation. Total RNA was extracted from the HM lipid fraction using the Norgen Circulating and Exosomal RNA Purification Kit (Norgen Biotech; Thorold, ON, Canada). The yield and quality of total RNA in each sample was assessed using the Agilent Bioanalyzer (Agilent Technologies; Santa Clara, CA, USA). Sample library construction and small RNA-sequencing occurred at the SUNY Molecular Analysis Core (State University of New York). Small RNA library preparation utilized 250 ng of RNA for each sample (Illumina Truseq Small RNA Prep Protocol), and underwent sequencing at a depth of 10 million, 50 base, paired-end reads per sample (NextSeq500 instrument, Illumina; San Diego, CA, USA). FASTQ files were deposited into the Gene Expression Omnibus repository (GSE192543). Mature miRNA reads were aligned to the human genome (hg38) using Bowtie2 and miRbase 22 in Partek Flow (Partek Inc.; Chesterfield, MO, USA) [22].

### 2.4. Infant Weight Data Collection

Infant weight and demographic data were extracted from the patients’ electronic medical records. Weight-for-length (WFL), weight-for-age, and length-for-age were converted to World Health Organization Z scores with the ‘anthro’ R package. Missing data (0.08 of WFL scores across infant ages) were imputed with k-nearest neighbor (KNN) row-wise imputation in MetaboAnalyst4 [28]. This machine learning approach allowed estimation of infant weight trajectory in cases where partial weight curves were available (due to missed well child visits) and prevented retention bias. For global context, obesity status was assigned with the World Health Organization criteria, but to compare within the study, infant obesity was considered as a 12-month WFL Z-score at or above the 95th percentile. Conditional weight gain (CWG) was calculated as the studentized residuals of a regression of 12-month weight-for-age Z score against birth weight-for-age Z score, birth and 12-month length-for-age Z score, age, and sex, following previously published methods [29,30].

### 2.5. Statistical Analysis

To compare HM miRNA profiles over the first year of life, statistical analyses were performed on miRNA counts from HM samples collected at birth, 1 month, and 4 months from 192 mother–infant dyads. Shifts in the miRNA profile was assessed with PERMANOVA (adonis; vegan R package) on Aitchison’s distance. Principal coordinate analysis was used to visualize center log-transformed miRNA profiles with the microViz and ggpubr R packages [31,32].

To determine if any singular miRNA was predictive of infant obesity status at 12 months, total HM miRNA consumption was calculated as previously described, expressed as mg and normalized with Box–Cox normalization [22]. The analysis focused on 163 mother–infant dyads who provided HM samples and longitudinal survey data, permitting estimation of miRNA consumption. To determine candidate miRNAs to test for predictive utility of infant obesity, all miRNAs were first tested for a significant change in overweight or obese infants. A linear regression was tested for each miRNA and associations with (1) CWG percentile at 12 months, and (2) WFL-Z percentile at 12 months. *p* values were corrected for multiple comparisons with the false discovery rate (FDR). Due to the large number of tests, no miRNA displayed significant associations after FDR correction. Therefore, candidate miRNAs were chosen if the unadjusted linear regression *p* value was <0.05. These candidate miRNAs were used in logistic regression to determine their efficacy in predicting infant obesity status at 12 months compared to demographic data. Physiologic relevance of candidate miRNAs was explored in miRPath (V4.0). All gene targets of candidate miRNAs were identified with the miRTarBase 2022 algorithm, and over-represented MSigDB gene sets were characterized based on cell type, using the genes union merging method and a Fischer’s exact test with false detection rate correction.

Demographic variables were chosen for their a priori associations with infant obesity. These included maternal race, maternal pre-pregnancy obesity status, private health insurance, breastfeeding status at 6 months, and gestational diabetes. Two models were compared, a “reduced” model comprising only demographic data, and a “full” model comprising demographic data and candidate miRNAs. A third model was constructed with demographic data and miR-224-5p. Potential outliers were assessed with Cook’s distance, and multicollinearity was checked with variance inflation factors. Receiver operator curves (ROC) were calculated with the pROC R package [33]. Logistic regression was conducted with the ‘glm’ function and the binomial family in R version 4.2.3. Figures were generated with the ‘ggpubr’ and ‘microViz’ R packages.

## 3. Results

### 3.1. Weight Outcomes

**Figure 1:**
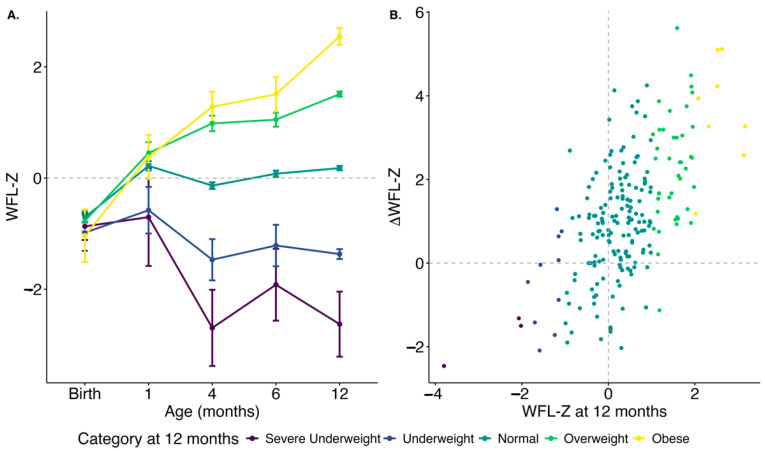
Weight-for-length Z scores across early growth, grouped by the infant obesity category at 12 months. (**A**) WFL of obese infants starts to differentiate around 4 months of age. (**B**) The change in WFL-Z from birth to 12 months (vertical axis; rapid weight gain) correlates to WFL-Z at 12 months.

At 12 months of age, 21 of the 221 infants (10%) had CWG over the 90th percentile and 30 of the 221 (13%) were at or above the 85th percentile. Most infants were born with a normal WFL Z-score [34] (WFL-Z; −0.746 ± 1.18) which increased with age; the average WFL-Z at 12 months was 0.404 ± 0.957 and at 24 months was 0.648 ± 1.03. When assigning obesity status with the World Health Organization WFL criteria at 12 months, 2 infants were severely underweight (2%), 9 were underweight (4%), 158 were normal (71%), 43 were overweight (19%), and 9 were obese (4%). In contrast, only seven infants were considered overweight and two obese at birth. Indeed, while most infants were born with a normal or underweight WFL-Z, by 4 months of age the infants’ WFL-Z became predictive of their 12-month obesity status (Figure 1A). Similarly, infants with rapid weight gain, measured by a positive change in WFL-Z at 12 months from birth, were more likely to have high WFL-Z at 12 months (Figure 1B). Taken together, this supports the premise that early growth dynamics can be predictive of obesity status at 12 months.

### 3.2. Human Milk miRNA Characteristics and Infant Weight Outcomes

**Figure 2:**
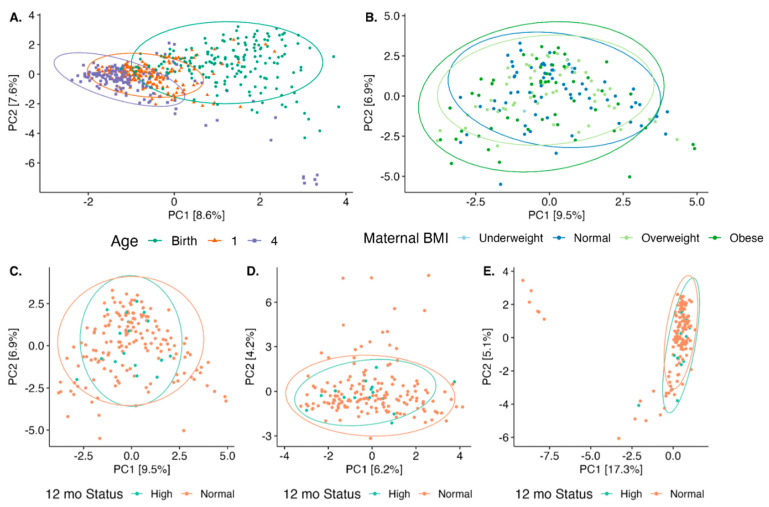
Principal component analysis of HM miRNA profiles (**A**) by infant age at the sampling time, (**B**) by mother’s pre-pregnancy obesity status, and by 12 month 95th percentile WFL-Z at (**C**) birth, (**D**) 1 month, or (**E**) 4 months.

A total of 1755 unique miRNAs were detected in HM. Multivariate analyses were conducted to determine candidate miRNAs for further testing in hierarchical logistic regression. The overall composition of HM miRNAs shifted from birth to 1 and 4 months (r^2^ = 6.4%, *p* = 0.001; Figure 2A). At birth, maternal pre-pregnancy obesity status did not drive HM miRNA composition (r^2^ = 1.5%, *p* = 0.87; Figure 2B). The overall HM miRNA profiles did not distinguish infant weight status at 12 months (95th percentile WFL-Z) when considering samples taken at birth (r^2^ = 0.6%, *p* = 0.4; Figure 2C), 1 month (r^2^ = 0.5%, *p* = 0.6; Figure 2D), or 4 months (r^2^ = 0.8%, *p* = 0.2; Figure 2E).

### 3.3. Logistic Regression of Demographic and miRNA Variables

Of the 221 mother–infant dyads enrolled in this study, 163 provided HM samples and survey details about infant feeding habits, enabling calculation of total miRNA consumption. Of these 163 mother–infant dyads, 15 had a corresponding CWG at or above the 95th percentile at 12 months of age. Linear regression of infant miRNA consumption with (1) CWG percentile at 12 months and (2) WFL at 12 months yielded a list of nine candidate miRNAs that were tested for their utility in predicting infant weight status (Table 1). Intriguingly, pathway analysis of these nine miRNAs showed that they target human cell types involved in glucose homeostasis and metabolism with greater frequency than expected by chance alone (Table 2). For example, 5 of the top 10 cell types targeted by the nine miRNA candidates arose from the pancreas, with additional cell types arising from the bile duct and liver.

**Figure 3:**
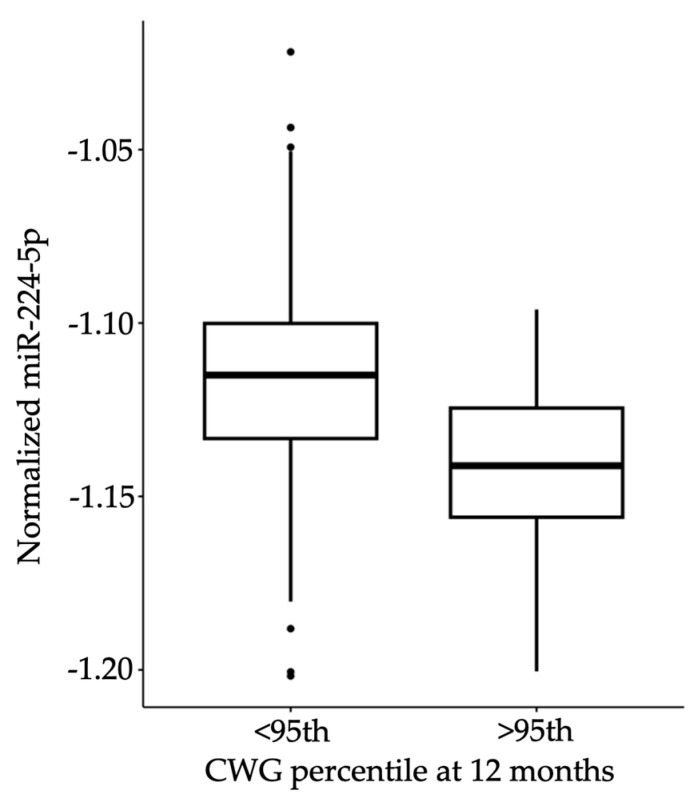
Consumption of HM miR-224-5p is significantly related to infant CWG status at 12 months. The boxplot shows the median and inter-quartile ranges with outlying points.

**Figure 4:**
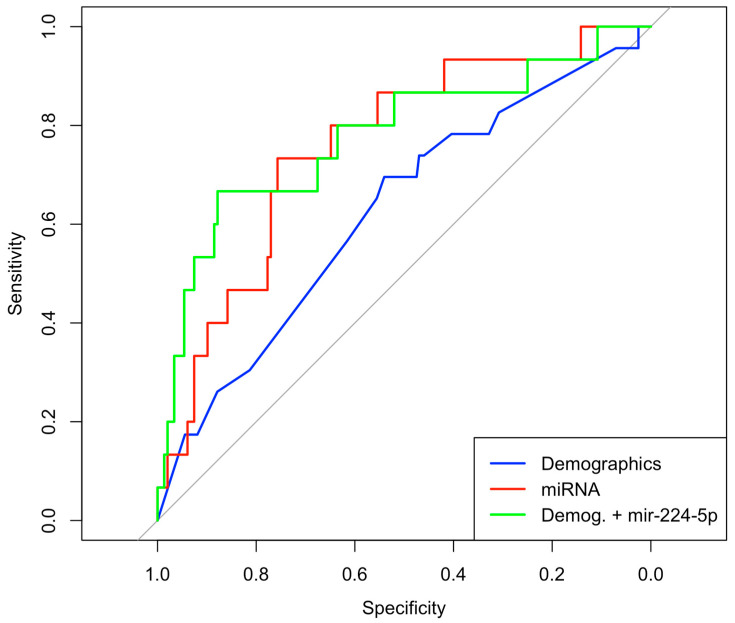
ROC curve showing the area under the curve (AUC) for three logistic regression models. The blue line shows AUC for predicting CWG at 12 months using only demographic variables (AUC = 62.4%); the red line shows prediction of CWG with only candidate miRNAs (AUC = 75.8%); and the green line shows prediction of CWG with demographic variables and miR-224-5p (AUC = 77.8%).

Logistic regression analysis with a reduced model tested only demographic information, including breast feeding status at 6 months of age, maternal ethnicity, presence of gestational diabetes, pre-pregnancy maternal BMI, and private health insurance (Akaike Information Criterion (AIC) = 155.7; Table 3). No coefficients were statistically associated with 12-month CWG, although the receiver operator characteristic (ROC) area under the curve (AUC) suggested fair predictive value of these demographic variables on CWG percentile at 12 months (AUC = 0.624). A logistic regression model using only candidate HM miRNA consumption to predict 12-month infant CWG (AIC = 108.75) yielded one significantly associated miRNA, miR-224-5p (Estimate −33.3, *p* = 0.014; Figure 3). The AUC of the model with only candidate miRNAs was 0.758. Because miR-224-5p was the only candidate miRNA to have a significant predictive value for infant obesity, we next tested its utility in predicting infant obesity when combined with demographic data. When a full model was constructed with miR-224-5p in addition to demographic variables (AIC = 98.16), only miR-224-5P remained a significant coefficient (AUC = 0.778, estimate = −31.42, *p* = 0.01; Figure 4).

## 4. Discussion

MiRNAs in human milk are bioactive factors tightly involved in postnatal epigenetic regulation, though their predictive power of early infant weight gain is unclear. Here, we explored the utility of HM miRNAs in predicting infant weight-for-length and conditional weight gain. These metrics of infant obesity were chosen based on the outcomes discussed in the INSIGHT trial [30]. Infant anthropometric data recapitulated previously published associations with early infant weight status. Infant WFL at 4 months was predictive of obesity category at 12 months. Further, infant growth rate during the first 12 months correlated with obesity category at 12 months. A previous study detected an association between WFL at birth and 6 months of life and obesity status at 3 years of age [5]. This suggests that there may be benefit to early educational and prevention programs, such as INSIGHT, the Healthy Beginnings Trial, and Nourishing Our Understanding of Role-modeling to Increase Support and Health (NOURISH), before 4 months of age [30,35,36].

Demographic information was shown to have weak associations with infant obesity. The regression model constructed with demographic factors including maternal race, maternal pre-pregnancy obesity status, private health insurance, breastfeeding status at 6 months, and gestational diabetes did not yield any significant coefficients, although the AUC suggested good predictive utility of these variables in predicting infant CWG. This is in contrast to other studies, which have shown that race, socioeconomic status [37], pre-pregnancy BMI [38,39], and maternal gestational diabetes [40] were associated or predictive of high infant WFL Z-scores. This may be due to homogeneity of these variables in the study cohort, which limits the power to detect differences in these demographic variables.

When analyzed together as a community, the entire HM miRNA profile shifted throughout the first four months of life and was not strongly affected by maternal pre-pregnancy obesity or infant obesity status at birth, one, or four months of age. This suggests that individual miRNAs may be involved in regulation of infant growth and metabolism but the miRNA profile is stable. Of the candidate HM miRNAs in the regression model, miR-224-5p was found to be significantly associated with CWG status at 12 months. miR-224-5p is involved in adipocyte differentiation and fatty acid metabolism [41]. Specifically, early growth response 2 (EGR2) and acyl-CoA synthetase long-chain family member 4 (ACSL4) are direct 3′ UTR targets of miR-224-5p. ACSL4 is an essential enzyme in fatty acid metabolism, which produces intermediates of complex lipids [42]. Interestingly, miR-224 was down regulated in peripheral blood mononuclear cells of obese participants in an energy-restriction weight loss treatment [43]. The differences in miR-224-5p consumption between normal-weight and overweight infants suggests that it may be involved in infant growth and development. Further studies are warranted to determine if miR-224-5p or its targets are candidates for interventions.

Together, this suggests a potentially limited role of HM miRNA in regulating infant rapid weight gain, although the utility of HM miRNA concentrations for predicting 12-month infant obesity in this cohort was weak. Larger study cohorts may be needed to detect subtle differences in miRNA profiles, or there may be additional influences of genetic background, diet, and environment that were not controlled for in this study. For example, studying HM miRNA associations with CWG in exclusively breastfed infants with familial risk for obesity may increase the power to detect an effect. The rate of infant obesity (12-month conditional weight gain over the 95th percentile) was approximately 10% in this study, limiting the power to detect differences between overweight and normal-weight infants. Future research should employ RNA spike-in controls and explore the role of miR-224-5p in adipogenesis to further understand the role it plays in metabolism and infant development and growth. It will also be important to validate these findings in an external cohort using quantitative polymerase chain reactions.

In conclusion, the results of this study contribute to our understanding of the factors influencing infant weight gain. These findings support prior studies demonstrating the predictive utility of rapid growth in early infancy for obesity-related outcomes. We identify one HM miRNA (miR-224-5p) that is biologically-related to adiposity and displays modest associations with infant weight trajectory. This molecule may play one small role among the wide variety of factors that regulate infant growth in the first year of life.

## Figures and Tables

**Table 1:** Candidate miRNAs with significant associations to 12-month infant CWG and/or WFL Z-scores. Each miRNA is shown with the linear model adjusted R^2^ and *p* value. Bolded entries meet statistical significance (*p* < 0.05).

miRNA	CWG R^2^ (*p* Value)	WFL R^2^ (*p* Value)
miR-30a-5p	4.7% (0.003)	2.3% (0.03)
miR-141-3p	4.1% (0.005)	1.5% (0.07)
miR-374b-5p	2.9% (0.02)	2.2% (0.03)
miR-29a-3p	2.7% (0.02)	1.0% (0.1)
miR-224-5p	1.3% (0.08)	2.6% (0.02)
miR-200a-3p	2.2% (0.03)	1.1% (0.09)
miR-151a-3p	1.6% (0.06)	1.8% (0.048)
miR-103a-3p	2.4% (0.03)	2.6% (0.02)
let-7i-5p	2.4% (0.03)	1.0% (0.11)

**Table 2:** Pathway analysis of candidate miRNAs displays over-representation of pancreatic cells. Cell type signature gene sets from MSigDB targeted by candidate miRNAs with greater frequency that expected by chance alone are shown, along with false detection rate (FDR)-corrected *p*-value on Fisher’s exact testing.

MSigDB Term	Genes Targeted, *n* (%)	FDR
Muraro Pancreas Ductal Cell	301/1522 (19.7)	1.25 × 10^−19^
Muraro Pancreas Mesenchymal Cell	160/716 (22.3)	2.01 × 10^−14^
Muraro Pancreas β Cell	195/1019 (19.1)	8.03 × 10^−11^
Aizarani Liver C39 Epcam Pos Bile Duct Cells	55/204 (26.9)	1.09 × 10^−7^
Hay Bone Marrow Immature Neutrophil	55/204 (26.9)	1.09 × 10^−7^
Muraro Pancreas Endothelial Cell	88/398 (22.1)	1.14 × 10^−7^
Lake Adult Kidney C17 Collecting System PCS Stressed Dissociated Subset	63/254 (24.8)	1.98 × 10^−7^
Cui Developing Heart C5 Valvar Cell	57/233 (24.4)	1.58 × 10^−6^
Aizarani Liver C20 LSECS 3	70/311 (22.5)	1.60 × 10^−6^
Muraro Pancreas Acinar Cell	151/851 (17.7)	2.41 × 10^−6^

**Table 3:** Summary of logistic regression model variables in the study cohort. Sample size is shown with the percentage of 221 mother–infant dyads enrolled in this study.

Model Variable	*n* (%)
Breastfeeding at 6 months	152 (69%)
Self-reported ethnicity (White)	166 (75%)
Presence of gestational diabetes	25 (11%)
Normal pre-pregnancy BMI	83 (38%)
Privately insured	167 (76%)

## Data Availability

The RNA sequencing data presented in this study are deposited in the Gene Expression Omnibus repository, accession number GSE192543. GEO repository link: https://www.ncbi.nlm.nih.gov/geo/query/acc.cgi?acc=GSE192543; accessed on 1 January 2022.
